# Evaluation of Halophyte Biopotential as an Unused Natural Resource: The Case of *Lobularia maritima*

**DOI:** 10.3390/biom12111583

**Published:** 2022-10-28

**Authors:** Anis Ben Hsouna, Monika Michalak, Wirginia Kukula-Koch, Rania Ben Saad, Walid ben Romdhane, Sanja Ćavar Zeljković, Wissem Mnif

**Affiliations:** 1 Laboratory of Biotechnology and Plant Improvement, Centre of Biotechnology of Sfax, University of Sfax, Sfax 3018, Tunisia; 2 Department of Environmental Sciences and Nutrition, Higher Institute of Applied Sciences and Technology of Mahdia, University of Monastir-Tunisia, Monastir 5000, Tunisia; 3 Collegium Medicum, Jan Kochanowski University, IX WiekówKielc 19, 35-317 Kielce, Poland; 4 Department of Pharmacognosy with Medicinal Plants Garden, Medical University of Lublin, 1 Chodzki Str., 20-093 Lublin, Poland; 5 Plant Production, College of Food and Agriculture Sciences, King Saud University, Riyadh 11451, Saudi Arabia; 6 Centre of the Region Haná for Biotechnological and Agricultural Research, Department of Genetic Resources for Vegetables, Medicinal and Special Plants, Crop Research Institute, Šlechtitelů 29, 78371 Olomouc, Czech Republic; 7 Centre of Region Haná for Biotechnological and Agricultural Research, Czech Advanced Technology and Research Institute, Palacky University, Šlechtitelů 27, 78371 Olomouc, Czech Republic; 8 Department of Chemistry, Faculty of Sciences and Arts in Balgarn, University of Bisha, Bisha 61922, Saudi Arabia; 9 ISBST, BVBGR-LR11ES31, Biotechpole Sidi Thabet, University of Manouba, Ariana 2020, Tunisia

**Keywords:** halophyte, *Lobularia maritima*, phytochemicals, stress genes, molecular mechanisms, biopotential

## Abstract

Halophytes are plant species widely distributed in saline habitats, such as beaches, postindustrial wastelands, irrigated lands, salt flats, and others. Excessive salt level, known to limit plant growth, is not harmful to halophytes, which have developed a variety of defense mechanisms allowing them to colonize harsh environments. Plants under stress are known to respond with several morpho-anatomical adaptations, but also to enhance the production of secondary metabolites to better cope with difficult conditions. Owing to these adaptations, halophytes are an interesting group of undemanding plants with a high potential for application in the food and pharmaceutical industries. Therefore, this review aims to present the characteristics of halophytes, describe changes in their gene expression, and discuss their synthesized metabolites of pharmacognostic and pharmacological significance. *Lobularia maritima* is characterized as a widely spread halophyte that has been shown to exhibit various pharmacological properties in vitro and in vivo. It is concluded that halophytes may become important sources of natural products for the treatment of various ailments and for supplementing the human diet with necessary non-nutrients and minerals. However, extensive studies are needed to deepen the knowledge of their biological potential in vivo, so that they can be introduced to the pharmaceutical and food industries.

## 1. Introduction

**Figure 1:**
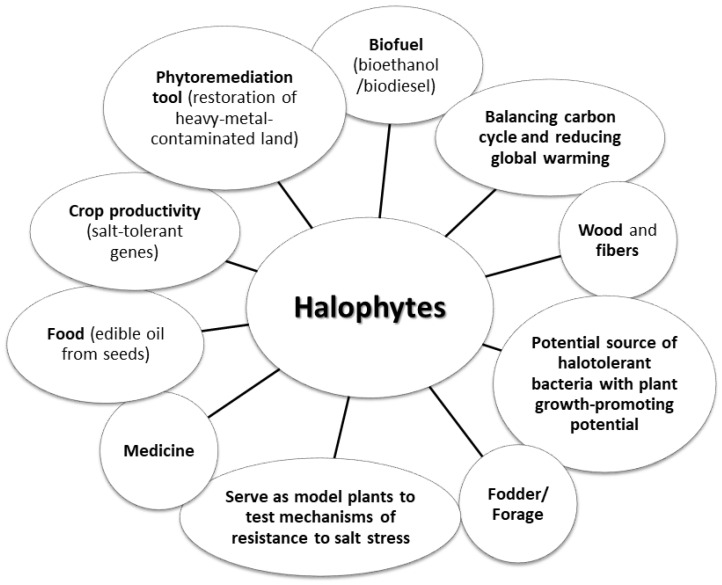
Potential use of halophytes (based [3,4]).

Halophytes are flowering plants that have adapted to soils with high salt concentrations and benefit from a salt-rich environment [1]. Plants with high salt tolerance represent just 2% of the world’s flora [2]. Salinization of the soil, which is undoubtedly one of the most important factors limiting plant growth [3], is a common occurrence, already affecting about 20% of the world’s agricultural land [4]. Interestingly, the total area of saline soils is increasing every year due to the formation of vast new areas of irrigated and cultivated lands, postindustrial wastelands, swamps, and lands in the vicinity of salty waters. In light of these trends, the studies on halophytes and their application in foods, medicine, and industry are gaining importance, as their ability to thrive on poor-quality water and soil makes them economically beneficial [3] (Figure 1, Table 1).

It is worth noting that plants under stress are known to develop a variety of defense mechanisms and to increase the biosynthesis of secondary metabolites enabling them to survive in a difficult environment. These substances can be of great importance to humans and can exhibit important biological functions [5] (Figure 1).

Therefore, this review aims to shed light on halophytes—species capable of surviving harsh environmental conditions—and to describe the Mediterranean plant *Lobularia maritima*, an example of a perennial, diploid (2 = 24) herbaceous halophyte of the family Brassicaceae, commonly known as sweet alyssum, in terms of its composition and pharmacological potential [6,7,8,9].

## 2. Salt Tolerance in Halophytes

Halophytes (salt-tolerant plants), unlike glycophytes (salt-sensitive plants), include plants that have adapted to complete their life cycle in the presence of high salt concentrations (≥0.2 M NaCl) [1,4]. Halophytes and glycophytes have similar salt response mechanisms, but the processes are differentially regulated [1]. Environmental stress tolerance mechanisms, including the modulation of photosynthesis, gas exchange, cell death, cell wall composition, cellular ion homeostasis, the transcription of stress-related genes, stress-protein synthesis, the generation of reactive oxygen species, the accumulation of secondary metabolites, antioxidant activity, and hormonal balance, involve several molecules and proteins encoded by stress-related genes [10,11,12]. Numerous genes of this kind have been isolated and functionally characterized among the representatives of halophytes, including *Salicornia brachiata* [13,14], *Aeluropus littoralis* [15,16,17,18], *Thellungiella halophila* [19,20,21], *Puccinellia tenuiflora* [22,23,24], *Phragmites australis* [25,26,27], and *Suaeda salsa* [28,29]. The above-mentioned genes encode transcription factors, signaling molecules, transmembrane proteins, osmoprotectants, Na^+^/H^+^ antiporters, potassium transporters, and antioxidative enzymes [30]. The molecular diversity and stress responses of halophytes are poorly studied; therefore, elucidating their tolerance mechanisms is important for application in other crops. Despite the complex nature of environmental stress tolerance mechanisms, master genes from halophytes have the biotechnological potential for crop improvement. These new crop varieties will help to meet the goal of a sustainable increase in global food production, minimize yield losses due to various environmental stresses, add value to food crops by fortification with vitamins, iron, carotenoids, anthocyanins, enhance the shelf life of fruits and vegetables, and stabilize food prices by ensuring a fluctuation-free assured food supply.

This section of the review article highlights the *Lobularia maritima* as a sample widespread halophyte, whose genes can have the potential for improving environmental stress tolerance in other plants or crops.

### 2.1. Lobularia Maritima Genes as Tools for Conferring Environmental Stress Tolerance to Crops

Halophytes are ideal models for elucidating the physiological, biochemical, and genetic mechanisms involved in alleviating cellular ionic imbalance and conferring salt tolerance [31]. Recently, several reviews have examined various aspects of halophyte physiology and discussed their potential applications in saline agriculture [30,31,32,33,34]; this has led to a renewed research interest in this area. To improve environmental stress tolerance in crop plants, it is necessary to identify candidate genes from halophytes to transfer them to salt-sensitive crops [35,36]. However, owing to the limited gene-sequencing information and challenges in identifying stress-related genes and their products, most halophytes are yet unsuitable for such studies. By contrast, the genomes of typical model glycophytes such as *Arabidopsis* sp. and rice, which have low stress tolerance, have been completely sequenced and their environmental stress response mechanisms extensively studied [37,38]. These sequencing data could facilitate deciphering stress tolerance mechanisms in closely related halophytes by comparative genomics. *Lobularia maritima* is an ideal halophyte model that meets these criteria [6,7,8]. Besides tolerating dry, poor, and polluted soils, *L. maritima* is a nickel hyperaccumulator that can remove different heavy metals from the soil [8]. *Lobularia maritima* is a facultative halophyte closely linked to *Arabidopsis thaliana* [39]; thus, it could be a suitable model for deciphering the molecular pathways underlying environmental stress tolerance in plants. Transcripts from *L. maritima* share 90% identity on average with homologous genes in *Arabidopsis* [40].

Many studies on *L. maritima* have focused on its maintenance, rapid in vitro multiplication, and methods of culture [8]. Despite the salt tolerance of *L. maritima*, it does not possess any salt-adapted morphological specializations (e.g., bladder cells or salt glands). Instead, its tolerance is attributed to adjustments in ionic and osmotic homeostasis [41]. Ben Hsouna et al. [42] demonstrated that *L. maritima* was a salt-tolerant halophyte that transported and accumulated Na^+^ in its shoots. Under salt stress, *L. maritima* successfully translocated Na^+^ from its roots while maintaining root K^+^ contents at levels similar to those in control plants [42]. Following this observed accumulation of ions, *L. maritima* exhibited a differential regulation of several Na^+^ and K^+^ transporter genes that are involved in maintaining ionic balance to survive high salt concentrations [39]. These findings confirm that *L. maritima* adapts to a high salinity and manages oxidative stress by rapidly developing efficient physiological and antioxidant mechanisms [42].

**Figure 2:**
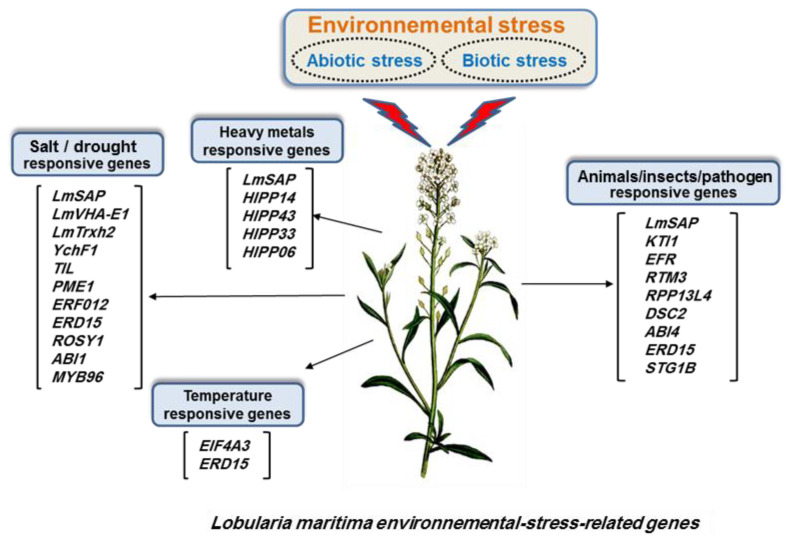
Environmental stress factors and main genes involved in adaptation and response in *Lobularia maritima*. Abbreviations: *LmSAP*: stress-associated protein; *LmVHA-E1*: vacuolar H+ -ATPase subunit E1; *LmTrxh2*: h-type Trx protein; *ABI*: ABA insensitive; *EFR*: EF-TU receptor; *EIF*: eukaryotic initiation factor; *ERD*: early responsive to dehydration stress; *ERF*: ethylene-responsive factor; *HIPP*: heavy-metal-associated isoprenylated plant protein; *KTI*: Kunitz trypsin inhibitor; *MYB*: myeloblastosis oncogene; *PME*: pectin methyl esterase; *ROSY:* interactor of synaptotagmin; *RPP*: resistance to *P. pachyrhizi*; *RTM*: restricted tobacco etches virus movement; *TIL*: temperature-induced lipocalin; *DSC2*: desmocollin-2.

Recent studies have shown that *L. maritima* genome is ~197.70 Mb in size, with 88.31% (174.59 Mb) of the sequences assigned to 12 pseudochromosomes [9]. The *L. maritima* genome is smaller than that of other Brassicaceae species; it contains 25,813 genes and large numbers of repetitive elements [9]. The adaptive divergence of a species from closely related species is frequently linked to gene families with significantly enlarged or contracted copy numbers [43]. The identification and isolation of novel salt-responsive genes and promoters from *L. maritima* should be explored for the potential genetic engineering of crop plants to enhance their abiotic stress tolerance using a transgenic approach. A better understanding of the salt response of *L. maritima* is necessary to exploit its potential as a source of stress-related genes. Several interesting genes with a demonstrated influence on salt tolerance have recently been isolated from this species. A total of 319 stress-related genes belonging to twenty-five gene families were found to be significantly enriched in *L. maritima* genome, which might have facilitated its adaptation to harsh environments [9]. These genes are primarily involved in the responses of *L. maritima* to (i) molecules of bacterial and fungal origin, (ii) insects, (iii) wounding stress, and (iv) heavy metals and abiotic stresses [9] (Figure 2). For example, the expanded *KTI* (Kunitz trypsin inhibitor) gene family comprises versatile protease inhibitors that are involved in the defense against insect attacks [44]. The *HIPP* (heavy-metal-associated isoprenylated plant protein) gene family is involved in responses to heavy metal stress [45], and *EIF4A3* (eukaryotic initiation factor) is important for abiotic stress adaptation and can partially regulate plant resistance to such stress by regulating the expression of acetoacetyl-CoA thiolase [46]. The *SGT1B* (suppressor of the G2 allele of skp1) gene is involved in the innate immunity and resistance of plants mediated by multiple R genes [47,48], wheras*YchF1* (an unconventional G protein) has been implicated in salinity stress tolerance and disease resistance against bacterial pathogens [49]. Dabbous et al. [50] described the complete isolation of *LmVHA-E1* gene (vacuolar H+ -ATPase subunit E1) from *L. maritima*. *LmVHA-E1* overexpression in *A. thaliana* led to improved tolerance to salinity and osmotic stress in transgenic plants, which was mainly associated with a reduced relative water loss and oxidative damages, and increased levels of sodium, possibly due to higher H+ -ATPase activity than that in the wild-type plants (WT). A recent study has revealed that the gene that encodes the h-type Trx protein—*LmTrxh2*—in *L. maritima* is more strongly induced in response to salt stress than to osmotic or oxidative stress, especially in the roots [51]. However, *LmTrxh2* overexpression in transgenic tobacco has been shown to enhance the overall tolerance of these plants to salt and osmotic stresses, possibly via the regulation of redox homeostasis [51].

Summarily, species-specific genes and expanded gene families may have promoted the adaptation of *L. maritima* to harsh environments, which is consistent with previous findings in numerous plants [52,53] (Figure 2). These genomic traits may explain why *L. maritima* hyperaccumulates nickel [8] and exhibits a high tolerance to salt stress [39].

### 2.2. Halophyte SAP Genes for Abiotic and Biotic Stress Response: A Well-Known Success Story

Over the last 15 years, interest in the A20/AN1 domain stress-associated protein (SAP) family (a class of zinc-finger proteins) has increased. These proteins exhibit structural and functional conservation among plant species [54,55]. In plants, SAP genes are induced by one or multiple abiotic stresses and function in a stress- and/or tissue-specific manner [54,56,57,58,59]. Recent studies suggest that SAPs act as ubiquitin ligases, redox sensors, and/or gene expression regulators under stress conditions [55,60,61,62,63,64,65,66]. Interestingly, the majority of the studies attempting to constitutively express SAPs in model plants (such as tobacco, *Arabidopsis*, and rice) report enhanced tolerance to multiple abiotic stress factors, including drought, salinity, cold, heat, oxidative stresses, and heavy metals [62,67,68,69,70].

Halophytes serve as a valuable source of adaptive genes. The A20/AN1 SAP gene, *AlSAP*, was first isolated from the halophyte grass *A. littoralis* [71]. The overexpression of this gene in transgenic tobacco [56], durum wheat [72], and japonica rice cv. Nipponbare [73] enhanced the plants’ tolerance to cold, drought, salinity, and oxidative stresses. Notably, *AlSAP* rice lines grown under drought stress during the reproductive stage exhibited higher yields than that obtained from the wild-type control, without incurring any yield penalty under irrigated field conditions [74]. Furthermore, the transcriptomic analysis performed by employing RNA-Seq technology using two *AlSAP*-expressor rice lines revealed a large number of deregulated stress-related genes [75]. This suggests that *AlSAP* transcript accumulation primes the expression of stress-related genes involved in transcription, signaling, protein degradation, and hormone homeostasis in rice plants [75]. Additionally, the upregulation of several negative pathogen defense regulators in *AlSAP* rice lines was associated with a low resistance to *Magnaporthe oryzae*.

**Figure 3:**
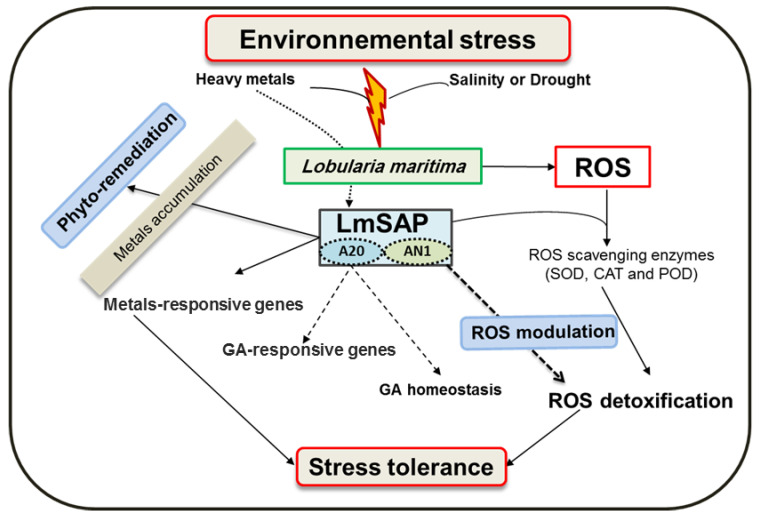
Hypothetical model for *LmSAP*-mediated abiotic stress tolerance via ROS modulation, GA homoeostasis, and heavy metals accumulation.

The second *SAP*-encoding gene isolated from the halotolerant plant *L. maritima* was designated *LmSAP* [57]. Its expression in *L. maritima* is induced by salt and ionic stresses, and its overexpression in transgenic tobacco plants enhances its tolerance to abiotic and heavy metal stress [65]. Ben Saad et al. [66] showed that *LmSAP* was involved in maintaining gibberellic acid (GA) homeostasis under abiotic stress conditions by regulating the expression of GA metabolism-related genes in transgenic tobacco, mainly via its A20 domain (Figure 3). Plants overexpressing *LmSAP*—full-length or truncated forms—that contained the A20 domain exhibited increased tolerance to salt and osmotic stresses, presumably via the positive modulation of the antioxidant genes expression involved in ROS scavenging and by reducing oxidative damage [76]. Additionally, *LmSAP* protects plant cells against oxidative stress by promoting ROS scavenging and by decreasing the intracellular concentration of free heavy metals (through its effect on metal-binding proteins in the cytosol) (Figure 3). Therefore, the *LmSAP* gene may be a potential candidate for introgression to crop plants to impart stress tolerance and phytoremediation.

Mishra and Tanna [30] argued that promoters from halophytes were promising candidates for genetic engineering since many stress-responsive genes are expressed in response to high stress. Thus, various cis-regulatory motifs of stress-responsive genes from halophytes have been examined in the last two decades [77,78,79,80,81]. The putative promoter region (1147-bp upstream of ATG) of the *LmSAP* gene (*PrLmSAP*) was also isolated from *L. maritima*, and its analysis revealed an active and organ-specific promoter induced by environmental stresses and wounding in transgenic rice [82]. Similar characteristics are exhibited by the promoter of the *AlSAP* gene, which shows an age-dependent activation, and its response to abiotic stress is induced in a tissue-specific manner [83,84]. To sum up, halophyte SAP genes represent a potential tool for engineering stress tolerance in crop species.

## 3. Phytochemical Composition of Halophytes

The results of studies on halophytes show that soil salinity affects not only the physiology of the plants, causing disturbances in their metabolism, development, and growth, but also the quality of plant material. Literature data indicate that salinity reduces the plant’s capacity for photosynthesis, which may decrease the total content of carbohydrates, fatty acids, and proteins [85]. On the other hand, in the conditions of salinity, plants have been observed to accumulate nitrogen-containing compounds such as amino acids (especially proline, but also alanine, arginine, glycine, serine, leucine, and valine), amides (such as glutamine and asparagine), polyamines, or specific classes of proteins (osmotin, dehydrins, and defensive proteins, i.e., late embryogenesis abundant (LEA) proteins) [86]. The concentrations of various secondary plant metabolites are strongly dependent both on the species and on the plant’s growing conditions, especially environmental conditions. The type of substances produced is species-specific [85,86]. In the scientific literature, there are contradictory reports on the changes in the phenolic compounds, chlorophylls, carotenoids, essential oils, and alkaloids content induced by salt stress [85,87]. Studies on various plant species show that the concentrations of phenolic compounds such as flavonoids, e.g., quercetin, apigenin [88], and anthocyanins [87], as well as phenolic acids, e.g., protocatechuic, chlorogenic, caffeic and *trans*-cinnamic acids [89], increase with soil salinity in plants. In the case of the essential oil constituents of various plants, the content of anethole [90], carvacrol [91], and eugenol [92] has been shown to decrease under saline conditions, while that of chamazulene, *α*-bisabolol, *trans*-*β*-farnesene [93], *γ*-terpinene [94] and linalool [92] increases. There is strong evidence that the content of photosynthetic enzymes, chlorophyll a, chlorophyll b, and total carotenoids decreases significantly as salinity increases [95].

### 3.1. Nitrogen-Containing Compounds and the Salt Tolerance of Halophytes

In general, halophytes adapt to increased salt levels via ionic compartmentalization, the production of osmolytes and compatible solutes, enzymatic changes, as well as the absorption of selective ions. The majority of these processes include the accumulation of nitrogen-containing compounds as a response to salt stress [96,97,98]. These compounds are mainly amino acids and polyamines, which are involved in various functions at cellular levels, such as salt excretion, maintaining the ion balance, the mitigation of oxidative stress, growth stimulation, favoring osmoregulation, etc. [99,100,101,102,103]. As stated by several authors [97,104,105], the content of nitrogen-containing compounds is mainly species-related, but still, the levels of the particular amino acids and polyamines increase significantly when plants are subjected to higher levels of salt. Kumari et al. [106] concluded that among amino acids, mainly proline, tyrosine, arginine, glycine, glutamine, and asparagine, together with the nonprotein amino acids, such as γ-aminobutyric acid, citrulline, and ornithine, are accumulated in halophytes under conditions of high salinity. In addition, polyamines, mainly putrescine, spermidine, spermine, and cadaverine, play a critical role in plants’ salt tolerance [106,107]. Polyamine biosynthesis starts from two amino acids, arginine and ornithine, which are decarboxylated to putrescine, a substrate for the synthesis of spermidine and spermine [96]. Moreover, spermidine and spermine serve as regulators of both nitrogen and carbon metabolism, causing the accumulation of other nitrogen forms such as amino acids glutamate, glutamine, and aspartate [108,109].

Since the understanding of the regulatory mechanisms acting on plant metabolic pathways is of great help to identify the potential candidates of plant metabolism under different environmental conditions, the evaluation and comparison of metabolite profiling data derived from the salt-sensitive (such as Sujala and MTU 7029 rice varieties) and salt-tolerant plant genotypes (such as Bhutnath, and Nonabokra rice varieties) can lead to the discovery of novel metabolites responsible for salinity tolerance in halophytes [110]. These authors suggest polyamines as the most promising compounds for acquiring resistance against salinity of their contribution not only to the protection of membranes, proteins, leaf water status, and cellular homeostasis but also to efficient biomass production under salinity conditions. Moreover, according to the available literature, it can be concluded that the manipulation of nitrogen metabolism is crucial for the understanding of plant salt tolerance. On the other hand, due to its complexity, the manipulation of plant metabolism is a difficult task. For example, the rate of amino acid and protein biosynthesis decreases in stressed plant cells, whereas protein degradation along with the accumulation of certain amino acids (e.g., proline) is highly induced [106,111]. Therefore, the manipulation of the amino acid and protein metabolism is not favorable because the homeostasis of these two processes is critical both under normal and stress conditions. As stated above, plants rich in polyamines usually show a strong salt tolerance [112]. Among them, spermidine and spermine seem to be the most important indicators [113,114,115]. However, Hernándiz et al. [116] recently showed that *Arabidopsis thaliana* seed priming with both putrescine and 1,3-diaminopropane showed an efficient level of salt tolerance for this salt-sensitive species. They found that the two polyamines induced glutamate metabolism, which was related to the synthesis of the polyamines. Moreover, the authors primed the seeds with ornithine to increase their tolerance to salt stress, but without any success. The reason for that might be in the activation of a ubiquitous strategy to better deal with stress or in the induction of negative stress (distress) in the plant. Therefore, the manipulation of polyamine biosynthesis seems to have the potential for the improvement of crop performance under salt stress. It is worth noting that due to the fluctuations in polyamine biosynthesis and their conversions, the timing and localization of the biosynthesis and conversion processes are crucial factors for this research [110].

Halophyte plants seem to be perfect models for studying polyamine metabolism, but still, there are just a few studies published on this topic that has been recently reviewed by Bueno and Cordovilla [98]. To the best of our knowledge, there is no literature review on polyamine profiles and/or contents of another halophyte: *Lobularia maritima*. However, Ben Hsouna et al. [42] found that proline content increased significantly when a plant was suffering salt stress, which is one of the plant strategies for its survival. Although this species was investigated thoroughly for its ability to survive severe abiotic stress conditions, there is still a lack of knowledge about its metabolome, especially nitrogen-containing compounds that might be responsible for managing salt stress. This is of high importance, especially for crops, whether they are halophytes or glycophytes. Ćavar Zeljković et al. [117] recently showed that the quality of basil and mint crops, defined by chemical composition and plant biomass, significantly changed when plants were grown in hydroponic solutions containing salt. Moreover, they found that stressed plants accumulated a polyamine—histamine. This fact significantly reduces plant quality, since histamine is an allergen to humans characterized by a low amine oxidase activity.

### 3.2. Active Components of Lobularia Maritima and Its Biological Properties

This section of the review highlights the chemical composition (Table 2) of *L. maritima* concerning its biological activity (in vitro studies).

Phytochemical screening indicates that *L. maritima* contains substances with high therapeutic value, such as fatty acids (capric, 9-oxononanoic, lauric, 14-methylpentadecanoic, palmitic, myristic, stearic, 16-methyloctadecanoic acid), terpenoids (dihydroactinidiolide, neophytadiene, betulinaldehyde, *β*-amyrin acetate, (+)-2-bornanone, and nootkaton-11,12-epoxide), and phytosterols (*β*-sitosterol, 24-methylenecycloartanol, and tremulone) [118,119,120]. In addition, many compounds accumulated by *L. maritima* function as antioxidants, eliminating reactive oxygen species, but are also responsible for the antibacterial, anti-inflammatory, anticancer, antiobesity, and hepatoprotective properties of the raw plants [86,118,119]. This group includes flavonoids, anthocyanins, phenolic acids, and tannins, as well as essential oil compounds and macromolecules such as proteins and polysaccharides [119,120,121,122].

An investigation of the phytochemical composition of *L. maritima* detected such phenolic compounds as 2,4-di-*tert*-butylphenol, vanillic acid, kaempferol, and quercetin derivatives [119]. Moreover, six acylated pelargonidin 3-O-sambubioside-5-O-glucosides have been found in the red-purple flowers of *L. maritima* [121]. GC/MS analysis showed that yellow essential oil obtained from the aerial parts of *L. maritima* contains 40 constituents, including oxygenated monoterpenes (74.40%) and monoterpene hydrocarbons (16.15%). The dominant components of the volatile oil are linalool (22.43%), benzyl alcohol (8.65%), 1-phenyl butanone (7.33%), *γ*-terpinene (6.15%), 1-terpineol (5.6%), *α*-cadinol (4.91%), globulol (4.32%), terpinen-4-ol (4.31%), a-terpineol (3.9%), ledol (3.59%), *α*-pinene (3.51%), and pulegone (3.33%) [122]. Heteropolysaccharides, composed of glucose, galactose, and xylose, with a molecular weight of 130.62 kDa, are interesting recently listed components of *L. maritima* [118].

#### 3.2.1. Antioxidant Activity

Scientific studies on halophytes often describe the role and the number of antioxidants that are capable of scavenging ROS in saline conditions [123]. Some reports show that plants with high levels of antioxidants, such as polyphenols, flavonoids, and vitamins, are much more resistant to salt stress [34,123]. Previous studies have shown that salt treatment (200 mM NaCl) significantly increased the phenolic and flavonoid content in the leaves of *L. maritima*, while a higher salinity (400 mM NaCl) caused a significant reduction in the content of these compounds. These results correlate with an antioxidant activity. The leaf extract of *L. maritima* displayed higher DPPH free-radical-quenching activity at 200 mM NaCl compared to the treatment with 400 mM NaCl [42]. In another study, the methanolic extract from the leaves of *L. maritima* was used for a compositional analysis and bioactivity studies [124]. The HPLC-DAD analysis revealed that the major constituents of the extract were gallic, salicylic, ellagic, ferulic acids, catechin, and quercetin, with salicylic acid as the leading molecule (120 mg/100 g DW). Thanks to the plentitude of phenolic components (total phenolic content: 175 ± 2.66 mg GAE/g DW, total flavonoids 35 ± 2.88 mg QE/g DW) the extract was found to be an active radical scavenger in in vitro tests (DPPH, lipid peroxidation inhibition test) [118].

The antioxidant activity of different parts of *L. maritima* from various geographic locations was evaluated using the 2,2-diphenyl-1-picrylhydrazyl (DPPH) colorimetric assay. DPPH scavenging potency was estimated at IC_50_ 5.16 to 9.33 mg/mL for the methanolic extract and 14.87 to 74.17 mg/mL for the aqueous extract of *L. maritima* aerial parts [125]. Another study showed that among various parts of the *L. maritima* plant, the root extract showed the highest capacity to scavenge DPPH free radicals, expressed as EC_50_ (0.08 mg/mL), which was comparable to the BHA standard (0.051 mg/mL). A lower antiradical activity was shown for extracts from the stem (3.9 mg/mL), flower (about 4 mg/mL), and leaf (4.2 mg/mL) [120]. Extracts from the aerial parts of *L. maritima* have also been shown to have good antioxidant properties in the *β*-carotene bleaching method, in which lipid substrates were used to determine biological activity [119].

Among plant secondary metabolites, essential oils and their constituents have also been shown to have antioxidant properties. These organic compounds (including terpene hydrocarbons and their oxygen derivatives, alcohols, aldehydes, and ketones) play an important role in scavenging free radicals and reducing oxidative stress due to the conjugated carbon double bonds and hydroxyl groups present in their structure, which can donate hydrogen [126]. The evaluation of the antioxidant properties of *L. maritima* essential oil has shown that monoterpene hydrocarbons, oxygenated monoterpenes, and/or sesquiterpenes may act as primary antioxidants. Research results showed that *L. maritima* essential oil exhibited strong radical scavenging activity compared to the standard ascorbic acid. In addition, the essential oil was shown to exert inhibitory effects on lipid peroxidation in the *β*-carotene bleaching method. Both the DPPH test and the *β*-carotene/linoleic acid bleaching test revealed that antioxidant activity increased in a dose-dependent manner [122].

An important group of plant-derived bioactive compounds that can regulate the redox state is polysaccharides. These high-molecular-weight polymers, composed of at least 10 monosaccharide molecules connected by glycosidic bonds, exert numerous beneficial biological effects, including antioxidant properties. Plant polysaccharides can be explored as a novel potential antioxidant, as they increase antioxidant enzyme activity, scavenge free radicals, and inhibit lipid peroxidation [126]. Studies concerning the antioxidant potential of crude polysaccharides from *L. maritima* showed that DPPH scavenging activity was proportional to the extract concentration. At high concentrations (300 μg/mL) the antioxidant properties of these macromolecules were higher than those of catechin at the same dose. Moreover, *L. maritima* polysaccharides presented the significant potential to inhibit C18:2 peroxidation, as well as moderate the reducing power, proportional to the polysaccharide concentration [118].

#### 3.2.2. Anti-Inflammatory Activity

Inflammation is a natural aspect of the immune system’s response to any type of damage. Irrespective of the route of activation and the factor inducing inflammation (including bacteria, viruses, parasites, or carcinogens), many proinflammatory mediators are released. These include numerous cytokines, such as interleukin 1*β* (IL-1*β*), interleukin 6 (IL-6), tumor necrosis factor α (TNF-*α*), and interferon *γ* (IFN-*γ*), which stimulate white blood cells, mainly macrophages, to produce large amounts of NO through the long-term activation of the enzyme nitric oxide synthase (iNOS). Another crucial enzyme for the inflammatory response, involved in the conversion of arachidonic acid to prostaglandins, is cyclooxygenase (COX). Similar to inducible NOS (iNOS), the most proinflammatory NOS isoform, COX-2 is recognized as the most active of the three known COX isoforms (COX-1, COX-2, and COX-3) during inflammatory processes [127,128]. Inflammation accompanies the development of many disorders and diseases, such as obesity, diabetes, cancer, atherosclerosis, and cardiovascular diseases. For this reason, researchers have been searching for new active substances with anti-inflammatory activity, including substances of natural origin [127,128]. In recent years, numerous articles have been published on the anti-inflammatory activity of plants and their secondary metabolites, e.g., polyphenols such as kaempferol, quercetin, rutin, luteolin, daidzein, genistein, and hesperidin, as well as alkaloids and terpenes [127,129]. Plants and their metabolites act by modulating induced iNOS and cells involved with inflammation, inhibiting the production of proinflammatory cytokines and modulating the activity of arachidic acid pathways, such as cyclooxygenase (COX), lipoxygenase (LOX), and phospholipase A2 [127,129].

In vitro studies have shown the inhibitory potential of *L. maritima* extract on NO production in lipopolysaccharide (LPS)-stimulated RAW264.7 macrophages [119]. Studies on the effects of *L. maritima* essential oil on inflammatory mediators in LPS-stimulated macrophages have shown that these bioactive compounds can regulate the expression of inflammatory cytokines. It was demonstrated that essential oil could modulate the inflammatory mode of macrophages by reducing levels of enzymes iNOS and COX-2 as well as proinflammatory cytokines IL-1*β*, IL-6, and TNF-*α* [122].

#### 3.2.3. Antiobesity

Obesity is associated with the development of many diseases, including diabetes, hypertension, osteoarthritis, cardiovascular diseases, and inflammation-based pathologies [130,131]. This is because adipose tissue is not only involved in energy storage but also functions as an endocrine organ secreting numerous bioactive substances known as adipokines. Most of them are proinflammatory, modulating the immune response agents that promote the development of metabolic diseases. Adipose tissue also produces pro-inflammatory cytokines such as TNF-*α*, IL-6, and IL-1*β* [131].

Literature data indicate that natural products can play a significant role as antiobesity drug candidates. Therefore, the search for new plant resources exhibiting potential health benefits is extremely important in the treatment of obesity. In the search for effective drugs, the role of polyphenolic compounds (such as quercetin, rutin, isorhamnetin, myricetin, hesperidin, and genistein) cannot be skipped, nor can the studies on their molecular mechanisms of action preventing and/or treating obesity [131]. The effects of polyphenolic compounds may rely on reducing the number of lipids absorbed from food products by inhibiting the activity of lipases. Therefore, one of the most commonly tested mechanisms for evaluating the potential efficacy of natural products as antiobesity agents is the inhibition of pancreatic lipase, a key enzyme for the absorption of dietary fats [119,130]. Inhibitory properties against pancreatic lipase have been confirmed for terpenes, saponins, and polyphenolic compounds, including flavonoids. Many plants have been screened for their potential to inhibit pancreatic lipase, including *Juniperus communis*, *Panax japonicus*, *Spilanthes acmella*, *Salvia officinalis*, *Glycyrrhiza uralensis*, *Vitis vinifera*, and others [130]. Marrelli et al. [119] conducted an in vitro study using a colorimetric method based on the use of 4-nitrophenyl caprylate as a substrate to confirm the potential antiobesity properties of *L. maritima*. The ethyl acetate polar fraction of *L. maritima* showed an inhibitory activity against pancreatic lipase, with an IC50 value of 1.33 ± 0.03 mg/mL [119].

#### 3.2.4. Antimicrobial Activity

Many studies have demonstrated the antimicrobial effects of plant extracts on various groups of pathogenic organisms. The antimicrobial properties of plants may be associated with their content of secondary metabolites such as flavonoids and tannins, as well as essential oils and their active components [120,132]. Phenolic compounds have been shown to exhibit a significant antimicrobial activity owing to the presence of the hydroxyl group. This group probably interacts with the cell membrane, disrupting its integrity and causing excessive leakage of metabolites and enzymes from the cell and a change in the lipid profile. This causes structural changes in the outer cell envelope and ultimately leads to a loss of viability [120]. Other known antimicrobial biomolecules include tannins, polyphenolic compounds containing hydroxyls, and other groups, such as carboxyls, which form strong complexes with various macromolecules [133].

Mechanisms explaining the antimicrobial activity of tannins include the inhibition of extracellular microbial enzymes, the deprivation of substrates required for microbial growth, or a direct action on the metabolism of microorganisms [120,133]. As in the case of phenolic compounds, terpenes act on the cell membrane, increasing its permeability. Terpenoids are known to affect the antibacterial properties of certain plants, possibly by influencing the nonmevalonate pathway. This pathway is very important in microorganisms (including Gram-negative bacteria and fungi) for the synthesis of cell membrane components and as a secondary carbon source [132]. Future investigations of plant material should focus on detailed phytochemical analysis and identifying correlations between secondary metabolites and antimicrobial activity.

Previous research has shown that *L. maritima* leaf, root, and flower extracts exhibit an antimicrobial activity against Gram-positive and Gram-negative bacteria (including *Staphylococcus aureus*, *Enterococcus faecalis*, *Pseudomonas aeruginosa*, and *Escherichia coli*) and fungi (*Aspergillus ochraceus* and *Aspergillus carbonarius*). The effective antimicrobial activity of *L. maritima* may be linked to the presence of tannins and flavonoids in different plant organs. The antimicrobial potential of different parts of *L. maritima* is also associated with compounds such as terpenoids, e.g., betulinaldehyde, neophytadiene, nootkaton-11,12-epoxide, menthol, (1*S*, 2*S*, 5*R*)-1′-(butyn-3-one-1-yl), and (+)-2-bornanone (camphor), as well as (*E*,*E*)-2,4-heptadienal, (*Z*)-9-octadecenamide, tributylacetylcitrate, and others. Most of the terpenes, including betulinaldehyde and menthol, responsible for the antibacterial and antifungal properties of *L. maritima*, were found to be present in the flower extracts. In addition, benzyl benzoate and 6-(methylsulfinyl) hexyl isothiocyanate were identified in the roots, while (*E*,*E*)-2,4-heptadienal, and 6-hydroxy-4,4,7a-trimethyl-5,6,7,7a-tetrahydrobenzofuran-2(4H)-one, also known as loliolide, was detected in leaf extracts [120].

Based on the literature and available research, it can be concluded that *L. maritima*, due to its content of bioactive compounds, could be a promising phytotherapeutic agent.

## 4. Pharmacological Properties of Halophytes with a Focus on *Lobularia maritima* (In Vivo Studies)

Halophytes are represented by a wide group of plant species that were proved to exhibit various pharmacological functions. Some of them are listed in the Table 3.

This section however focuses on a detailed analysis of the pharmacological profile of *Lobularia maritima.* This plant has been traditionally used in the Mediterranean region as a diuretic, antiscorbutic, antioxidant, and anti-inflammatory agent [153]. Vast applications of the plant in traditional medicine were certainly influenced by the presence of polyphenols and polysaccharides in the extract. The major indication for its use were urinary problems, including bladder and kidney infections, lithiasis, or prostate inflammation [153]. A 30-day-long therapy based on one cap of a whole plant infusion was recommended, e.g., in Algeria, and was found to show a detoxifying, diuretic, and antispasmodic action [153].

According to Ben Hsouna and coauthors [118], heteropolysaccharide isolated from *L. maritima* was found to exhibit protective properties toward the liver in the CCl_4_-induced hepatotoxicity model in Wistar albino rats. The molecule was characterized by a molecular weight of 130.62 kDa and when hydrolyzed, it was found to contain glucose, xylose, fructose, rhamnose, mannose, and galactose moieties. The administered compound significantly decreased the secretion of liver enzymes (AST, ALT, ALP, and LDH) whose levels in the control group were elevated due to the intoxication process. Moreover, a marked antioxidant potential of the polysaccharide was noted in several assays. The authors reported that the tested compound protected the liver against oxidative stress damage. In addition, the tested molecule induced the secretion of IL-10 in the rat serum, which explained its anti-inflammatory potential and the ability to reverse the toxic effects of CCl_4_ (observed downregulation of TGF-*β*1 and TNF-*α*). Furthermore, upon the administration of the polysaccharide, the liver (analyzed postmortem) did not show any signs of fibrosis. Moreover, when tested on Wistar male rats in a similar assay as reported previously [118], 500 mg/kg b.w. of the tested sample showed a statistically insignificant 21% decrease in the levels of liver enzymes (AST/GOT) and (ALT/GPT) in comparison with the CCl_4_-exposed group. However, a marked elevation of the catalase (CAT), superoxide dismutase (SOD), and glutathione peroxidase (GPx) levels was described in the alyssum-treated group in comparison to the intoxicated animals. Furthermore, a moderate correction of the CCl_4_-induced damage to the liver was visualized in histological analysis in the group supplemented with *L. maritima*. The applied dose was not toxic to the animals.

The flowers of sweet alyssum are rich sources of volatile components that can exhibit different functions. The major components of the essential oil investigated for its in vivo effects were described as linalool benzyl alcohol 1-phenyl butanone γ-terpinene α-cadinol and others [122]. The above-described oil was found to exhibit similar protective properties as previously described polysaccharides isolated from the plant or methanolic extract from the leaves of *L. maritima*. The essential oil induced the production of antioxidant enzymes in CCl_4_-treated rats, at a dose of 250 mg/kg b.w., modulated the response of the immunologic system by reducing iNOS and COX-2 enzymes together with IL-1*β*, IL-6, TNF*α*, and reduce the expression of cytokines responsible for the liver inflammation (Table 4).

The essential oil obtained from the flowering parts of the plant is commonly used as a repellent to protect crops from deterioration. In their study, Renkema and Smith [154] described a reduction in the number of attracted *Drosophila suzukii* flies to raspberries—most probably thanks to the presence of acetophenone and benzaldehyde among the volatiles of the plant. The repellent and insecticidal properties of the essential oil from *L. maritima* were also described by Wang and colleagues [155] in an assay with three-grain pests: *Sitophilus oryzae*, *Tribolium castaneum*, *and Callosobruchus maculatus*. Thanks to the presence of *trans*-3-pentenenitrile, a strong fumigant effect of the oil was observed especially against *C. maculatus*. A 100% repellency was noted for the concentrations of 0.05 and 0.1 nL/cm^2^ against *C. maculatus* and *S. orizae*, respectively, and 93% against *T. castanum* (0.2 nL/cm^2^).

The above examples of its application confirm the anti-inflammatory, antioxidant, antispasmodic, detoxifying, and repellent properties of the plant and its constituents. However, more studies related to the in vivo application of *L. maritima* should be performed soon to complete its activity profile in animal tests as the plant is abundantly studied in in vitro models. These tasks are very important, concerning the fact that *L. maritima* is regarded as an edible and safe plant.

## 5. Conclusions

Based on the review it can be concluded that halophytes are important sources of primary and secondary metabolites of different kinds. They were proven to contain chlorophylls, polyphenols, terpenes, saponins, fatty acids, and other groups of natural products. The extracts obtained from halophytes (e.g., from *Lobularia maritima*) exhibited important biological properties, e.g., antimicrobial, anticancer, anti-inflammatory, detoxifying, and weight-reducing action. Having in mind the cited references, the majority of studies were however performed in in vitro tests and the anti-inflammatory action was the only one proved in in vivo test on animals. Therefore, it is important to pursue more detailed studies on living organisms to add novel results to the existing research.

## Figures and Tables

**Table 1:** Examples of use of selected species of halophytes [3,4].

Use	Species
Crops	*- Lobularia maritima* *- Aeluropus littoralis* *- Populus euphratica* *- Karelinia caspica* *- Suaeda salsa* *- Kalidium foliatum* *- Puccinellia tenuiflora*
Food	*- Lobularia maritima* *- Suaeda fruticosa* *- Arthrocnemum macrostachyum* *- Halopyrum mucronatum* *- Cressa cretica* *- Haloxylon stocksii* *- Alhaji maurorum*
Medicine	*- Lobularia maritima* *- Enicostema verticillatum* *- Haloxylon stocksii* *- Parkinsonia aculeata*
Fodder/forage	*- Aeluropus logopoides* *- Atriplex stocksii* *- Chenopodium album* *- Panicum turgidum* *- Desmostachya bipinnata* *- Salvadora persica* *- Sporobolus helvolus* *- Tamarix indica* *- Urochondra setulosa*
Biofuel	*- Desmostachya bipinnata* *- Phragmites karka* *- Halopyrum mucronatum* *- Panicum turgidum* *- Typha domingensis*

**Table 2:** Important bioactive compounds in *Lobularia maritima* [118,119,120,121,122,123,124,125,126].

Compound		Key Biological Properties
Phenolic compounds	TP (mg GAE/g): *-* flowers 60.845 - leaves 147.451 - stems 307.873 - roots 368.150	- classified as primary antioxidants - eliminate radicals through direct reactions, scavenging, or reduction of free radicals (e.g., hydroxyl, superoxide, peroxide, and alcoxyl radicals) to less reactive compounds - chelate transition metal cations (e.g., Cu2^+^ and Fe2^+^) - inhibit the activity of many enzymes involved in free-radical generation (e.g., xanthine oxidase, protein kinase and lipoxygenase) - exhibit anti-inflammatory, antibacterial, antifungal, antiviral, antiallergic, anticancer, anticoagulant, and astringent properties
	TF (mg CE/g): - leaves 0.432 - roots 0.346 - flowers 0.088 - stems 0.021	
	TC (mg CE/g): - stems 0.310 - flowers 0.303 - leaves0.195 - roots 0.109	
Fatty acids	e.g., capric, lauric, palmitic, myristic, stearic acid	- important cell membrane components - precursors of eicosanoids (PG, PGI, TX, LT), tissue hormones with a broad spectrum of activity - exhibit anti-inflammatory and antiallergic effects - activate metabolic processes and cell division
Phytosterols	e.g., *β*-sitosterol	- play structural roles in cell membranes - reduce cholesterol and LDL-C plasma levels - exert antiatherogenic effects
Terpenoids	e.g., neophytadiene, betulin aldehyde, *β*-amyrin	- exhibit effective activity against various bacterial, fungal, and yeast strains - exhibit anti-inflammatory activity - exert anticancer effects
Essential oil compounds	e.g., linalool, benzyl alcohol, 1-phenyl butanone, 1-terpineol	- exhibit antiseptic, antimicrobial, antifungal, anti-inflammatory, immunostimulatory, neuroprotective, and antioxidant properties
Macromolecules	e.g., proteins, polysaccharides	- important organic components of the body - exhibit antioxidant properties through a variety of mechanisms, including free-radicals scavenging, electron or hydrogen transfer reduction, transition-metal-chelating activity, ferric reducing power, and prevention of LPO

TP, total phenols; TF, total flavonoids; CT, condensed tannins; GAE, gallic acid equivalent; CE, catechin equivalent; PG, prostaglandins; PGI, prostacyclin; TX, thromboxanes; LT, leukotrienes; LDL-C, low-density lipoprotein cholesterol; LPO, lipid peroxidation.

**Table 3:** Selected biological properties of halophytes.

Species	Botanical Family	Properties	Reference
*Rubia tinctorum*	Rubiaceae	Diuretic action; treatment of type II diabetes mellitus	[134,135]
*Tamarix gllica*	Tamarixaceae	Astringent, antibacterial, anti-inflammatory, wound-healing, and diuretic properties	[136]
*Limoniastrum monopetalum*	Plumbaginaceae	Cardioprotective, antidysenteric, antioxidant, antidiarrheal properties	[137,138]
*Verbena officinalis*	Vebenaceae	Analgesic, anti-inflammatory, anticancer, neuroprotective, and anticonvulsant activity	[139]
*Plantago lanceolata, P. major, P. ovata*	Plantaginaceae	Anticancer, anti-infectious, anti-inflammatory action in hepatitis; treatment of cold, cough, and digestive disorders	[140,141]
*Teucrium genus*	Labiatae	Antispasmodic, hypoglycemic, anti-inflammatory, analgesic properties	[142]
*Caesalpinia crista*	Leguminosae	Treatment of headaches, cough, asthma, neurodegenerative diseases, and upset stomach	[143,144]
*Terminalia catappa*	Combretaceae	Preventing hepatoma, hepatitis, fever, and diarrhea	[145,146,147]
*Cakile maritima*	Brassicaceae	Diuretic, antiscorbutic, anti-inflammatory, purgative and digestive properties	[148,149]
*Salsola kali*	Amaranthaceae	Hypotensive, hypoglycemic, anticancer, procognitive, antiviral, antimicrobial, hepatoprotective properties	[150]
*Inula viscosa*	Asteraceae	Antiseptic, antiscabies, antipyretic, anticancer, anti-inflammatory agent	[151,152]

**Table 4:** Pharmacological properties of *Lobularia maritima* in the light of in vivo studies.

Compound	Organism	Administration	Dose	Duration	Action Mode	Reference
Heterpolysaccharide	Male Wistar albino rats	i.p.	250 mg/kg b.w.	15 days	Anti-inflammatory, detoxifying ↓ ALT ↓ AST ↓ ALP ↓ LDH ↑ SOD ↑ CAT ↑ GPx ↑ IL-10 ↓ TGF-β1 ↓TNF-α	[118]
Methanolic extract from the leaves	Male Wistar albino rats	p.o.	100–500 mg/kg b.w.	30 days	Anti-inflammatory and detoxifying ↓(AST/GOT) ↓ (ALT/GPT) ↑ SOD ↑ CAT ↑ GPx	[124]
Whole plant infusion	Traditional medicine	p.o.	One cup on an empty stomach	30 days	Treatment of urinary problems Antiradical, anti-inflammatory, diuretic properties	[153]
Essential oil	Male albino Wistar rats	i.p.	250 mg/kg b.w.	15 days	Anti-inflammatory properties, antioxidant properties ↓ IL-1β ↓IL-6 ↓ TNF-α ↓iNOS ↓COX-2 ↓Inflammatory cytokines	[122]

i.p., intraperitoneal administration; p.o., oral administration; b.w., body weight.

## Data Availability

Not applicable.
